# NonHodgkin's Lymphoma with Peritoneal Localization

**DOI:** 10.1155/2014/723473

**Published:** 2014-03-09

**Authors:** E. Curakova, M. Genadieva-Dimitrova, J. Misevski, V. Caloska-Ivanova, V. Andreevski, B. Todorovska, U. Isahi, M. Trajkovska, P. Misevska, N. Joksimovic, S. Genadieva-Stavric, S. Antovic, N. Jankulovski

**Affiliations:** ^1^University Clinic of Gastroenterohepatology, Medical Faculty, Ss. Cyril and Methodius University, Mother Teresa 17, 1000 Skopje, Macedonia; ^2^University Clinic of Hematology, Medical Faculty, Ss. Cyril and Methodius University, Mother Teresa 17, 1000 Skopje, Macedonia; ^3^University Clinic of Digestive Surgery, Medical Faculty, Ss. Cyril and Methodius University, Mother Teresa 17, 1000 Skopje, Macedonia

## Abstract

The gastrointestinal tract is the most common extranodal site involved with lymphoma accounting for 5–20% of all cases. Lymphoma can occur at any site of the body, but diffuse and extensive involvement of the peritoneal cavity is unusual and rare. We report a case of diffuse large B-cell lymphoma in a 57-year-old female infiltrating the peritoneum and omentum and presenting with ascites and pleural effusion. The performed examinations did not discover any pathological findings affecting the digestive tract or parenchymal organs, except for diffuse thickening of the peritoneum and omentum. Peripheral, mediastinal, or retroperitoneal lymphadenopathy was not registered. The blood count revealed only elevated leukocytes and on examination there were no immature blood cells in the peripheral blood. The cytology from the ascites and pleural effusion did not detect any malignant cells. Due to the rapid disease progression the patient died after twenty-two days of admission. The diagnosis was discovered postmortem with the histological examination and immunohistochemical study of the material taken during the surgical laparoscopy performed four days before the lethal outcome. Although cytology is diagnostic in most cases, laparoscopy with peritoneal biopsy is the only procedure which can establish the definitive diagnosis of peritoneal lymphomatosis.

## 1. Introduction

Extranodal lymphoma occurs in about 40% of all patients with lymphoma and has been described in virtually every organ and tissue [1]. Extranodal disease is more common with nonHodgkin's lymphoma (NHL); it is often intermediate- to high-grade [2, 3] and the extranodal involvement is in general a poor prognostic factor [4]. Secondary involvement of extranodal tissue as part of generalized lymphoma is significantly more common than primary extranodal disease in which there is a dominant extranodal component with no or minor nodal involvement [4]. The gastrointestinal tract is the most common extranodal site involved with lymphoma accounting for 5–20% of all cases and a gastrointestinal involvement is usually secondary to widespread nodal disease [5]. Diffuse large B-cell lymphoma (DLBCL) and follicular lymphoma are the dominant histological subtypes in extranodal lymphoma [4]. Although primary gastrointestinal lymphomas (PGLs) are relatively rare, they are still the most common type of primary extranodal lymphomas. PGL accounts for 40% of all extranodal NHLs, for 4%–20% of all NHL cases, and for about 1%–4% of all gastrointestinal malignancies [4, 6–9]. Almost 90% of the PGLs are of B cell lineage and of the nonHodgkin type [10]. Lymphoma can arise from any region of the gastrointestinal tract, but the stomach is the most commonly involved site followed by the small intestine and the ileocecal region [11].

DLBCL is the most common histological NHL subtype in adults accounting for approximately 25% of all NHL cases [12, 13]. An extranodal involvement occurs in about 40% of the DLBCLs [14, 15]. DLBCL is also the most common pathological type of gastrointestinal lymphoma in essentially all sites of the gastrointestinal tract [10] and the gastrointestinal DLBCL is the most frequent extranodal lymphoma [16]. In the Western world, nearly 90% of aggressive mature B-cell lymphomas are identified as DLBCL [17]. DLBCL is a heterogeneous disease that displays different clinical, histological, immunophenotypic, cytogenetic, and molecular features, suggesting that in fact the DLBCL spectrum includes several different disease entities [18]. Based on clinical, morphological, immunological, and genetic features the World Health Organization (WHO) divides DLBCL into many different variants, subgroups, and subtypes (Table 1) [19, 20]. Patients with DLBCL typically present with nodal (most usually cervical or abdominal) or extranodal disease and they usually exhibit rapid tumor growth and symptoms highly dependent upon the tumor localization [17]. Tumor cell origin, B symptoms, and International Prognostic index are important clinical predictors of survival in primary gastrointestinal DLBCL [21, 22]. DLBCL carries a poor prognosis and if left untreated takes an aggressive and fatal clinical course with a median survival of less than one year [23]. Despite the new treatment protocols, for certain subgroups of patients the clinical outcomes are still unsatisfactory [17].

Although lymphoma can occur at any site of the body, diffuse and extensive involvement of the peritoneal cavity is rare. Multiple intra-abdominal organ infiltration or disseminated peritoneal lymphoma, called peritoneal “lymphomatosis” (PL), receives much less attention in the literature than peritoneal carcinomatosis, probably due to its relative infrequency [24, 25]. The patterns of peritoneal involvement include smooth peritoneal thickening, discrete nodules, a diffuse infiltrative mass without symptoms of bowel obstruction, and presence of nonloculated and nonseptated exudative ascites with high protein content [4, 26, 27]. DLBCL presenting with PL is rare, but there are cases where DLBCL can occur as a form of PL without solid tumor component [28]. PL is usually associated with high-grade lymphoma and aggressive histological subtypes and it is considered to be an aggressive presentation of lymphoma [29, 30].

## 2. Case

**Figure 1:**
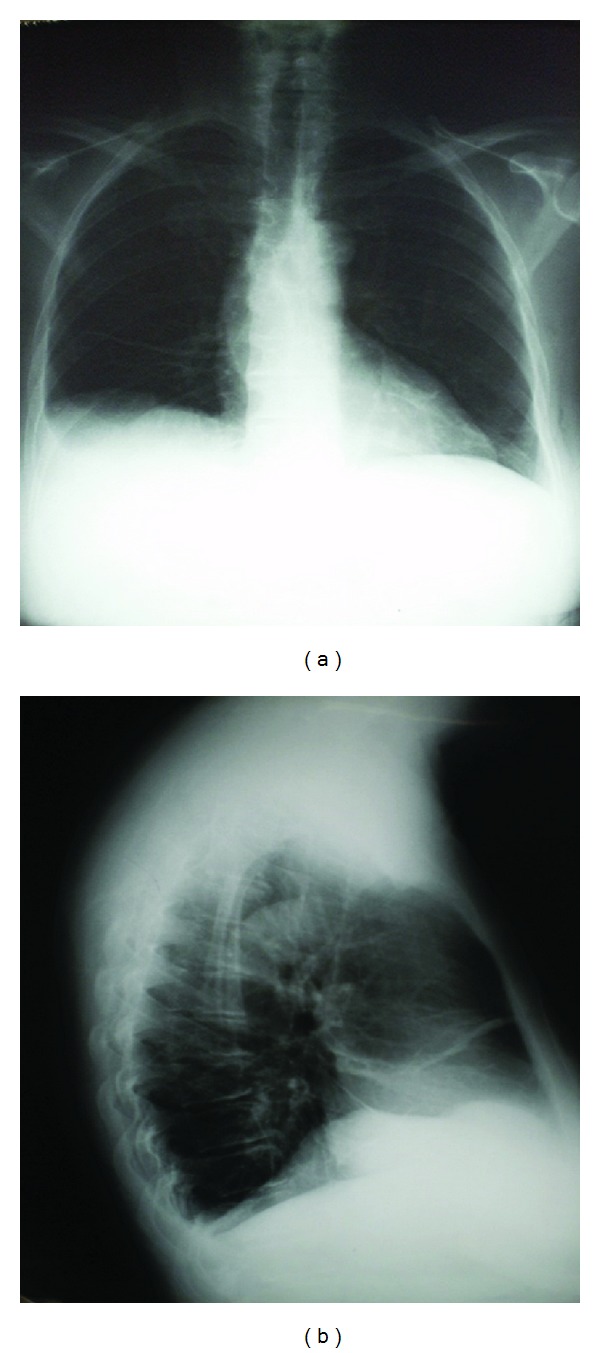
A 57-year-old woman with diffuse large B-cell lymphoma and peritoneal lymphomatosis chest radiography shows right-sided pleural effusion without mediastinal enlargement or other significant abnormalities.

**Figure 2:**
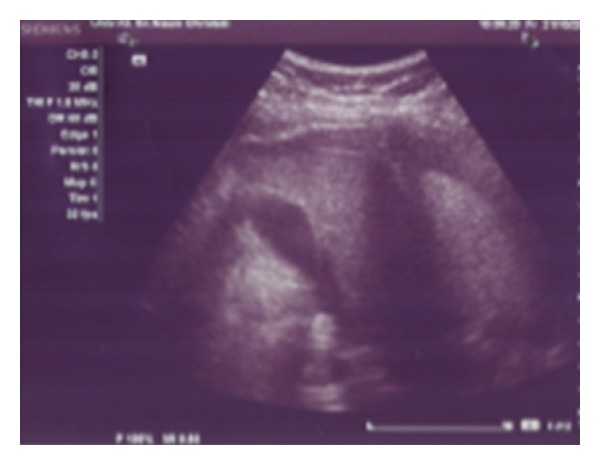
Abdominal ultrasound shows ascites without findings of liver cirrhosis or portal hypertension.

**Figure 3:**
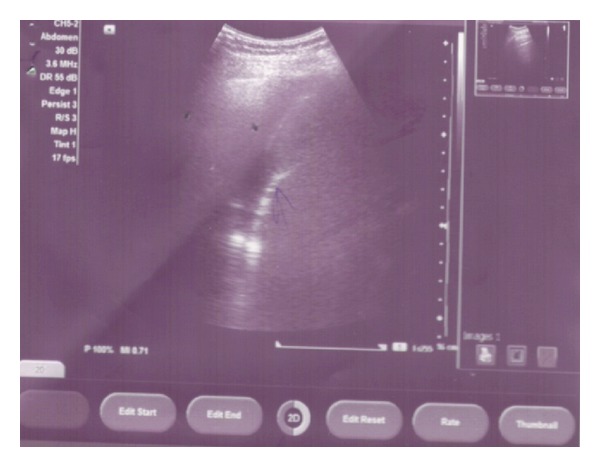
Thoracic ultrasound shows effusion in the right pleural space.

**Figure 4:**
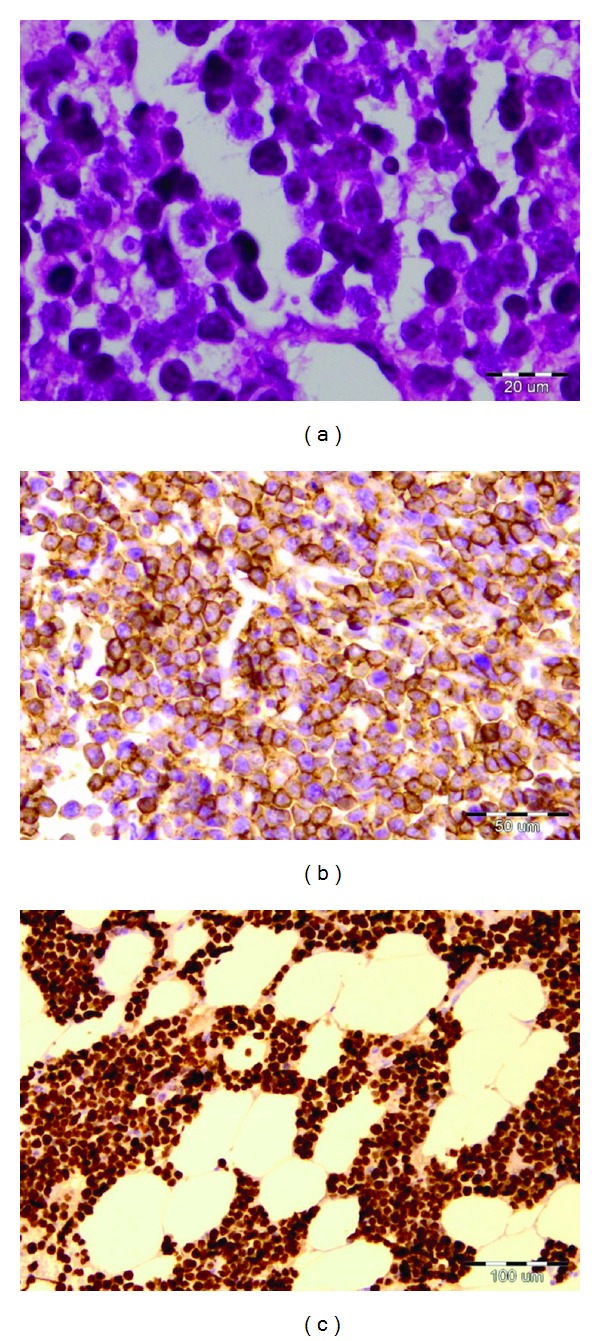
Histological features from peritoneal and omental biopsies. H&E stain: (a) diffuse infiltration of neoplastic lymphoid cells (400x). (b) Tumor cells immunoreactive for B cell marker CD 20 (200x). (c) Tumor cells expressing high Ki-67 index (100x).

A 57-year-old woman was admitted to the Clinic of Gastroenterohepatology with a two-month history of general weakness, malaise, abdominal fullness, increased abdominal gear, nausea, vomiting, and peripheral edema (Figure 1). No fever was noted before admission. She had a distended and dull abdomen without peritoneal signs and her bowel sounds were normal. There were no enlarged and palpable lymph nodes. The patient did not have significant previous medical history. Eight years ago she underwent hysterectomy with bilateral oophorectomy due to uterine myoma.Two years ago the patient had a total colonoscopy in another medical institution, but no remarkable abnormalities were found. One year ago she had a total body technetium scan which detected few accumulations in the cervical spine interpreted as lesions of degenerative origin. The peripheral blood count was initially unremarkable (hemoglobin 134 g/L, hematocrit 41%, red blood cells count 4.5 × 10^12^/L, white blood cells count 9.8 × 10^9^/L, and platelet count 197 × 10^9^/L). During the stay the leukocyte count raised up to 16.7 × 10^9^/L with granulocytic predominance. The peripheral blood smear showed no immature cells (80.4% neutrophils, 8.5% lymphocytes, 10.7% monocytes, 0.1% eosinophils, and 0.3% basophils). The routine laboratory tests showed no specific abnormalities, except for the remarkable LDH values of 3194 U/L (normal value: 213–423 U/L) and uric acid of 1068 *μ*mol/L (normal value: 150–450 *μ*mol/L). The total plasma protein level was 58 g/L, albumin level 35 g/L, globulin level 23 g/L, and CRP level 37 mg/L. Renal and liver functional tests, bilirubin, and electrolytes were normal. The serological screening for B and C hepatitis (HBsAg and anti-HCV) was negative. The abdominal ultrasound showed large amount of ascites without findings of liver cirrhosis or portal hypertension (Figure 2). The CT scan revealed diffusely thickened, nodular, and irregular peritoneum mainly affecting the upper parts of the abdomen and the anterior abdominal wall, findings which are initially consistent with peritoneal carcinomatosis. The CT scan did not indicate any tumor formation, bowel affection, or abdominal lymph node enlargement. The diagnostic paracentesis yielded chylous fluid. The LDH level in the peritoneal fluid was extremely elevated reaching values of 32585 U/L and the LDH ascites/serum ratio was 0.05. The protein level was ranging from 28 to 39 g/L, albumin level from 13 to 26 g/L, globulin level from 7 to 13 g/L, glucose level 0.2 mmol/L, cholesterol level 2 mmol/L, and triglycerides level 1.5 mmol/L. Serum-ascites albumin gradient ranged from 14 to 16 g/L. The tumor markers were also analyzed. The serum carbohydrate antigen 125 (CA 125) level was 597.9 U/mL (normal value <35.0 U/mL) and the ascites CA 125 level was elevated up to 4221 U/mL. The levels of carcinoembryonic antigen (CEA), CA 19–9 and CA 72–4 were within the normal range. The adenosine deaminase (ADA) level in ascites ranged from 67.5 U/L to 122.9 U/L and ADA ascites/serum ratio ranged from 3 to 5.91. Lysozyme level ranged from 11.2 mg/L to 20.7 mg/L. The chest radiography and thoracic ultrasound revealed small unilateral pleural effusion (Figure 3). The pleural fluid was hemorrhagic and the biochemical analysis showed LDH level of 175.8 U/L (LDH pleural fluid/serum ratio 0.05), total protein of 24.2 g/L, albumin 15.5 g/L, glucose 0.3 mmol/L, CRP 19.8 mg/L, triglycerides 0.4 mmol/L, and cholesterol level of 1.5 mmol/L. The ADA and lysozyme values in the pleural fluid were 43.4 U/L and 9.0 U/L, respectively, with pleural fluid/serum ratio of 1.95 for ADA and 0.76 for lysozyme. The tumor markers in the pleural fluid were not elevated. Because of the elevated ADA in ascites, we performed additional investigations (acid-fast staining, the Lowenstein-Jensen culture test of ascitic fluid, and the Mantoux test) in order to exclude peritoneal tuberculosis, but all these findings were negative. The gastroscopy showed few small mucosal elevations with central depression. The cytology from the peritoneal and pleural fluid did not detect any malignant cells (cytology class I) with massive coagulation necrosis, granulocytes, and macrophages in the ascitic fluid. The diagnostic laparoscopy performed by a gastroenterologist discovered only thickened peritoneum, but the histological analysis of the peritoneal biopsies did not reveal the nature of the findings. Afterwards a surgical laparoscopy was performed which also confirmed diffuse peritoneal thickening and nodular omental infiltration. During the diagnostic process the patient became febrile up to 38.5°C and she developed progressive cardiorespiratory deterioration which led to multiorgan failure and lethal outcome within twenty-two days of admission despite all symptomatic and supportive measures. The definitive diagnosis was established postmortem with the histological report from the peritoneal biopsies taken during the surgical laparoscopy four days before the lethal outcome. The preliminary histological report unexpectedly showed presence of neoplastic lymphoid cells diffusely infiltrating the fibrous and fat tissue with “starry sky” phenomenon, findings which are consistent with aggressive type of B-cell NHL (Figure 4). Afterwards, immunohistochemical study was performed and showed CD 20+ expression with proliferative fraction higher than 80% in favor of DLBCL. The patient's family did not allow us to perform an autopsy; hence we were not able to discover any other potential lymphomatous involvement affecting other organs and tissues and to determine the disease extension and stage.

## 3. Discussion 

Isolated PL is a rare condition and there are only several similar case reports and autopsy reports published. Aslam presented an autopsy report of a high-grade T-cell NHL confined to the peritoneum, mesentery, omentum, and surfaces of abdominal viscera, without deeper extension into the underlying parenchyma or other primary localizations [31]. We present an unusual case of DLBCL with peritoneal lymphomatosis without other obvious primary involvements. The patient did not have any specific symptoms or physical findings and the laboratory data were inconclusive. The thickened peritoneum with deposits accompanied by elevated ascitic LDH and CA 125 strongly suggested carcinomatosis. Because of the elevated ascitic ADA, tuberculous peritonitis was also suspected, but the additional test did not confirm the diagnosis. The blood count and peripheral blood smear were almost normal and since we did not register peripheral or abdominal lymph node enlargement, we practically did not suspect any lymphoproliferative disease. The process of getting the definitive histological diagnosis in our case was difficult, complex, and time consuming. Confronting many different laboratory data that were inconsistent, confusing, and sometimes conflicting, the imaging features and the CT scan findings were the most concrete features and they served as a framework for our diagnostic process. Imaging findings showing diffusely thickened and nodular peritoneum and nodular omental infiltration with massive ascites were initially misleading towards peritoneal carcinomatosis. PL occurs less frequently than peritoneal carcinomatosis and most of the previously reported cases were also initially misdiagnosed for metastatic carcinoma [24, 28, 29, 32]. Because the omentum lacks lymphoid elements, lymphomatous omental infiltration is uncommon and the route of this dissemination is not completely clear. The presumed way of propagation is believed to be via the pathways like gastrocolic ligament, transverse mesocolon, and visceral peritoneal surface [33]. Lymphomatous omental infiltration is mostly reported in NHL and in only few Hodgkin's lymphoma cases [34]. As many reports also suggest, patterns of tumor involvement of peritoneum, omentum, and mesentery seen in PL are mostly indistinguishable from those seen in peritoneal carcinomatosis [31, 35, 36]. PL closely mimics peritoneal carcinomatosis and it is almost impossible to differentiate peritoneal carcinomatosis from lymphomatosis based on imaging findings only, especially in cases where the peritoneal infiltration is the only evident involvement and no other findings compatible with lymphoma exist. In many of the reported cases the cytology was able to detect lymphoid cells in the peritoneal fluid and to establish the diagnosis [24, 25, 28, 37]. It seems that the cytology of ascites is a simple and usually effective method for making a diagnosis from adequate samples with time limitation [24]. However, lymphoma can evoke florid mesothelial hyperplasia which can result in a confusing cytology result and extend the diagnostic process [31, 32]. In a series of seven patients with NHL and PL, that Lynch et al. report, the cytology was diagnostic in only one patient [26]. Cases with inconclusive cytology and/or histology findings, especially when carcinoma and carcinomatosis are erroneously suspected, require laparotomy in order to establish the diagnosis [29, 30, 38]. In advanced and rapidly progressive lymphoma cases with significantly affected general condition, an unnecessary laparotomy or erroneous tumor debulking procedure may not be the best option since it can contribute to additional progression and deterioration [24, 29, 30].

It is well known that the serum LDH is elevated in many lymphoma forms and in primary gastrointestinal lymphoma the LDH level at diagnosis seems to be an independent prognostic factor [21, 39]. But despite the well-established relation between lymphoma and serum LDH, there are no clinical studies which define the relationship between PL and elevated ascites LDH levels [28]. Besides the remarkable serum LDH values of 3194 U/L, we also registered an extremely elevated ascites LDH level of 32585 U/L, much higher than in the few other PL cases [24, 28], which suggests that ascites LDH level is well elevated in PL and could be an important clue for differential diagnosis [28].

CA 125 is a mucin-like glycoprotein antigen expressed in normal tissue originally derived from celomic epithelium such as peritoneum, pleura, pericardium, fallopian tubes, and endometrium [40]. Although it can be considered to be a sensitive marker for ovarian epithelial neoplasm, it still has a limited specificity. CA 125 can be elevated in other malignancies (carcinoma of the endometrium, breast, lungs, or pancreas) or different benign conditions (endometriosis, uterine leiomyoma, cirrhosis with or without ascites, pelvic inflammatory disease, and in pleural or peritoneal fluid from any reason) [41–45]. A few studies and sporadic case reports have reported an elevated CA 125 levels in patients with low grade and in aggressive NHL [46, 47]. In most studies the serum CA 125 levels ranged from 1070 to 1400 U/mL especially in patients with effusion [46, 48]. In our patient we registered moderately elevated serum CA 125 (597.9 U/mL), but the CA 125 level in the ascites was remarkable reaching 4221 U/mL. High CA 125 level in lymphoma is associated with advanced disease stage, poor performance status, pleural or peritoneal fluid, high LDH, mediastinal and/or abdominal involvement, elevated International Prognostic Score, partial or absent response of treatment, and with decreased survival independent of the stage of the disease [40, 46, 48–50].

ADA is a purine-degrading enzyme necessary for maturation and differentiation of lymphoid cells. ADA activity of ascitic fluid has been proposed as a highly sensitive (100%) and specific (97%) nonculture method of detecting tuberculous peritonitis especially when using cut-off values from 36 to 40 IU/L [51]. Considering these values, elevated ADA in ascites of 122.9 U/L and ADA serum/ascites ratio of 5.91 in our patient raised the suspicion for specific peritonitis, but the repeated and additional tests did not confirm diagnosis. ADA can be elevated in different noninfectious conditions associated with pleural and peritoneal fluid lymphocytosis, including malignancies (adenocarcinomas, leukemias, and lymphomas) and connective tissue diseases (rheumatoid arthritis, systematic lupus erythematosus) [24, 52]. However, elevated ADA in ascites has rarely been reported in the diagnosis of lymphoma [53]. False negative and false positive ADA values are possible, and therefore an elevated ADA in ascites should be interpreted in relation to the overall clinical presentation and should not be considered equivalent to the presence of mycobacteria [52, 54].

There are only few cases in the literature where the lymphomatous involvement of the peritoneum and omentum is the only localization of lymphoma. Mostly, the peritoneal lymphomatous affection is secondary to continuous spread from some other part of the gastrointestinal tract [24, 29, 37, 38] or from abdominal lymph nodes [32, 33]. Lymphoma constitutes 15%–20% of all small intestine neoplasms and 20%–30% of all primary gastrointestinal lymphomas with ileum being the most common site (60%–65%) [10, 55]. The pattern of small-bowel involvement includes solitary or multiple nodules and circumferential wall thickening with or without aneurysmal dilatation [56, 57] and on the contrary of gastrointestinal adenocarcinoma, lymphoma is more likely to involve multiple and longer bowel segments [58]. Small bowel lymphoma mostly presents with abdominal pain, nausea, vomiting, weight loss, and bowel dysfunction (protein losing enteropathy and/or malabsorption syndrome) and less frequently with intussusceptions or gastrointestinal bleeding [37, 59, 60]. Bowel obstruction is less probable and unusual and bowel perforation is also uncommon. [56–58]. DLBCL is the most common histological subtype of NHL that occurs in large bowel with frequency ranging from 47% to 81% depending on the geographic location [61–63] and within the large bowel cecum and rectum are the most common sites. The most common symptoms are abdominal pain, weight loss, changes in bowel habits, and gastrointestinal bleeding [62–65]. Also, in more than half of the patients lymphoma is a bulky disease reaching over 5 cm in diameter [63–65] which usually makes it easily palpable by simple physical examination and detectable by abdominal ultrasound [63].

In our case, gastroscopy with biopsy excluded primary lymphoma affecting the proximal GI tract. In order to evaluate the large bowel, colonoscopy was also planned, but this was prevented by the patient's general condition and the tendency for further deterioration. It should be emphasized that in all of the reported cases of intestinal lymphoma with diffuse peritoneal infiltration and massive ascites, the CT scans suggested some form of bowel affection [24, 29, 38, 40]. In cases of intestinal lymphoma the CT scan usually detects focal or diffuse circumferential bowel wall thickening, a stenotic bowel segment with or without aneurismal dilatation, encasement of the small-bowel wall, poor delineation at the mesenteric border, lymph node enlargement, massive tumor formation, or abdominal mass with external bowel compression [24, 26, 29, 36, 38, 40]. Our patient did not report significant bowel dysfunction symptoms and the CT scan did not detect any of the previously described radiological findings suggesting primary bowel involvement. Despite these facts, without an autopsy report, we cannot exclude a case of primary intestinal lymphoma infiltrating the peritoneum with certainty.

Primary effusion lymphoma (PEL), formerly known as “body cavity-based lymphoma,” is a rare NHL type limited to body cavities without detectable tumor formation. According to the WHO classification of hematopoietic malignancies (Table 1), PEL is classified as a distinct subtype of DLBCL and is directly and universally associated with human herpesvirus-8. Epstein-Barr virus (EBV) and human immunodeficiency virus (HIV) have also a certain role in the pathogenesis of PEL [66]. Although most PELs occur in HIV-infected patients, several cases have been described in immunodeficient HIV-negative patients, such as in transplant recipients and patients with cancer or cirrhosis [67]. PEL is characterized by its unique predilection for serosal surfaces, including the pleura, pericardium, and peritoneum [68]. Most affected patients present with a symptomatic serous effusion containing high-grade, malignant lymphocytes and the dominant symptoms, such as dyspnea or abdominal distension, are usually related to mass effect from fluid accumulation [68]. Chest radiographs and CT scans reveal pleural and/or pericardial effusion, slight serosal thickening in absence of detectable solid mass lesion [69, 70], and parenchymal abnormalities or mediastinal enlargement [69–71]. The diagnosis of PEL is usually made on a cytological preparation of the involved effusion fluid and due to the unique liquid phase of growth of the tumor the samples are almost always positive for malignant cells [69, 71]. The malignant cells contain the genomic material from HHV-8 and the detection of the HHV-8 in the nuclei of the PEL cells is a key diagnostic criterion. The aggressive nature of PEL results in a resistance to conventional chemotherapy and poor prognosis with a short median survival of only 6 months [72]. The intense disease progression, the diagnostic delay, and the unperformed autopsy precluded additional investigations and more specific immunohistochemical and genetic analyzes in order to confirm or exclude any other potential primary lymphomatous involvement and to define the lymphoma subtype more precisely. Still, taking into account everything previously stated, PEL could also be a possible option.

## 4. Conclusion

Peritoneal lymphomatosis is considered a rare manifestation of aggressive histological types of high-grade lymphomas, characterized by systemic involvement and rapid clinical deterioration often rapidly leading to death. Therefore, it is extremely important to provide correct and early diagnosis in order to provide treatment and prolong survival.

In lymphoma cases surgical intervention can contribute to disease progression and additional clinical deterioration. Image-guided needle biopsy or laparoscopic peritoneal and omental biopsy appears to be the gold standard method for diagnosing PL. Therefore, they should be used in order to make the diagnosis preoperatively and every effort should be made to avoid unnecessary laparotomy or massive surgery whenever possible.

Gastrointestinal lymphoma and PL have a variable and nonspecific presentation and can resemble other neoplastic or inflammatory conditions. Clinical presentation, imaging spectrum, or laboratory data alone can be inconclusive and misinterpretations of the findings could initially presume incorrect diagnosis, extend the diagnostic process, and delay the diagnosis which reduces the possibility for optimal treatment. Therefore, only focused clinical awareness, high level of suspicion, and complementary approach can lead towards accurate diagnosis.

## Figures and Tables

**Table 1:** DLBCL variants, subgroups, and subtypes (from Mey et al. [17]).

DLBCL, not otherwise specified	
Common morphologic variants:	
Centroblastic	
Immunoblastic	
Anaplastic	
Rare morphologic variants	
Molecular subgroups	
GCB	
ABC	
Primary mediastinal large cell lymphoma	
Immunohistochemical subgroups	
CD5-positive DLBCL	
GCB-like	
NonGCB-like	

DLBCL subtypes	

T-cell/histiocyte-rich large B-cell lymphoma	
Primary DLBCL of the CNS	
Primary cutaneous DLBCL, leg type	
EBV-positive DLBCL of the elderly	

Other lymphomas of large B-cells	

Primary mediastinal (thymic) large B-cell lymphoma	
Intravascular large B-cell lymphoma	
DLBCL associated with chronic inflammation	
Lymphomatoid granulomatosis	
ALK-positive LBCL	
Plasmablastic lymphoma	
Large B-cell lymphoma arising in HHV8-associated multicentric Castleman's disease	
Primary effusion lymphoma	
